# An Optimised Human Cell Culture Model for Alveolar Epithelial Transport

**DOI:** 10.1371/journal.pone.0165225

**Published:** 2016-10-25

**Authors:** Hui Ren, Nigel P. Birch, Vinod Suresh

**Affiliations:** 1 Auckland Bioengineering Institute, University of Auckland, Auckland, New Zealand; 2 School of Biological Sciences, University of Auckland, Auckland, New Zealand; 3 Centre for Brain Research, University of Auckland, Auckland, New Zealand; 4 Brain Research New Zealand, Rangahau Roro Aotearoa, New Zealand; 5 Department of Engineering Science, University of Auckland, Auckland, New Zealand; 6 Maurice Wilkins Centre for Molecular Biodiscovery, University of Auckland, Auckland, New Zealand; Universidad de la Laguna, SPAIN

## Abstract

Robust and reproducible *in vitro* models are required for investigating the pathways involved in fluid homeostasis in the human alveolar epithelium. We performed functional and phenotypic characterisation of ion transport in the human pulmonary epithelial cell lines NCI-H441 and A549 to determine their similarity to primary human alveolar type II cells. NCI-H441 cells exhibited high expression of junctional proteins ZO-1, and E-cadherin, seal-forming claudin-3, -4, -5 and Na^+^-K^+^-ATPase while A549 cells exhibited high expression of pore-forming claudin-2. Consistent with this phenotype NCI-H441, but not A549, cells formed a functional barrier with active ion transport characterised by higher electrical resistance (529 ± 178 Ω cm^2^ vs 28 ± 4 Ω cm^2^), lower paracellular permeability ((176 ± 42) ×10^−8^ cm/s vs (738 ± 190) ×10^−8^ cm/s) and higher transepithelial potential difference (11.9 ± 4 mV vs 0 mV). Phenotypic and functional properties of NCI-H441 cells were tuned by varying cell seeding density and supplement concentrations. The cells formed a polarised monolayer typical of *in vivo* epithelium at seeding densities of 100,000 cells per 12-well insert while higher densities resulted in multiple cell layers. Dexamethasone and insulin-transferrin-selenium supplements were required for the development of high levels of electrical resistance, potential difference and expression of claudin-3 and Na^+^-K^+^-ATPase. Treatment of NCI-H441 cells with inhibitors and agonists of sodium and chloride channels indicated sodium absorption through ENaC under baseline and forskolin-stimulated conditions. Chloride transport was not sensitive to inhibitors of the cystic fibrosis transmembrane conductance regulator (CFTR) under either condition. Channels inhibited by 5-nitro-1-(3-phenylpropylamino) benzoic acid (NPPB) contributed to chloride secretion following forskolin stimulation, but not at baseline. These data precisely define experimental conditions for the application of NCI-H441 cells as a model for investigating ion and water transport in the human alveolar epithelium and also identify the pathways of sodium and chloride transport.

## Introduction

The alveolar lining fluid is a very thin liquid layer which is essential for maintaining efficient gas exchange, surfactant homeostasis, and defence against inhaled toxins and pathogens [1]. Ion and water transport across the alveolar epithelium regulates the depth and composition of the liquid layer. The basic mechanism of fluid transport is well established: vectorial transport of Na^+^ and Cl^-^ between the apical (air-facing) and basolateral (blood-facing) surfaces establishes an osmotic pressure gradient that results in net water movement between the alveolar and interstitial spaces [1]. However, under disease conditions such as acute lung injury (ALI), the transport process is disrupted, which results in the accumulation of edema fluid and impairment of gas exchange [2].

The alveolar epithelium is composed of type I and II pneumocytes. Equipped with a great number of epithelial junctions and ion-transporting proteins, they control the balance of the alveolar fluid layer. First of all, type I and II cells express junctional proteins such as E-cadherin, claudins, occludin and zona occludens (ZO) [3–5]. These junctions seal the paracellular clefts between neighboring cells, serving not only as a mechanical barrier, but also a determinant for the paracellular permeability and selectivity to water and different ions. The specific protein composition of epithelial junctional complexes defines the barrier characteristics and generates tight or leaky epithelium [3, 5]. Type I and II cells also express various channels, transporters, and pumps for Na^+^, Cl^-^ and water transport. The major pathway for Na^+^ transport across the alveolar epithelium is through the apical epithelial Na^+^ channel (ENaC) and the basolateral Na^+^-K^+^-ATPase transporters [6]. Concurrent Cl^-^ transport parallel to Na^+^ transport maintains electrical neutrality. It was initially thought that Cl^-^ moved passively through the paracellular pathway, but the importance of channels and co-transporters is now well established [1, 7]. Of these, the cystic fibrosis transmembrane conductance regulator (CFTR) is the principal pathway at the apical membrane although other Cl^-^ channels such as voltage-gated and calcium-activated chloride channels may also contribute. Electroneutral cotransporters (Na^+^-K^+^-2Cl^-^ and K^+^-Cl^-^) and exchangers (HCO_3_^-^-Cl^-^) constitute the basolateral transcellular pathway. The water transport proteins aquaporin-3 (AQP3) and aquaporin-5 (AQP5) are expressed in the alveolar epithelium [8] and are considered to facilitate osmotically-driven water transport across the apical membrane [9]. However, studies in AQP knockout mice did not affect fluid clearance or edema formation suggesting that their functional significance for water transport in the alveoli is limited [9, 10]. These studies point to the ongoing evolution in our understanding of alveolar fluid transport.

Cell culture models have provided important information regarding the rate, direction and regulation of transport since they offer the ability to characterise and perturb individual proteins and pathways under tightly controlled conditions. While primary human cells are the most representative of the *in vivo* situation, few studies have used them [11, 12] since they are not widely available and lose their functional properties upon passaging [13]. A recent study has successfully passaged human primary alveolar epithelial type II cells up to two generations while retaining their phenotype and functional properties, but the availability of the source material remains a bottleneck [14]. Human embryonic stem cells, induced pluripotent stem cells and mesenchymal stem cells have also been successfully differentiated into alveolar epithelial cells with type II cell markers and functional surfactant uptake and release [15–17]. The results offer promise for the development of *in vitro* models that more closely correspond to *in vivo* tissue, but as yet fluid and ion transport have not been characterised in these models. A number of studies have used primary cells from various animal species [13], but species-specific differences in fluid transport rates and sensitivity to agonists and inhibitors may limit applicability to humans [2]. Respiratory epithelial cell lines of human origin could offer alternative *in vitro* models that are robust and reproducible. Two human pulmonary epithelial cell lines, A549 and NCI-H441, have been widely used as models for the alveolar epithelium for biopharmaceutical research [18–23]. A549 cells were isolated from a human pulmonary adenocarcinoma and exhibit a phenotype similar to that of type II cells due to the presence of lamellar bodies and surfactant proteins [20, 21, 24]. NCI-H441 cells were originally isolated from the pericardial fluid of a patient with papillary adenocarcinoma of the lung and show characteristics of both type II and club cells [20, 25]. A number of studies have identified ENaC and Na^+^-K^+^-ATPase expression in these cell lines as the primary pathway for sodium transport and characterised their function and regulation [26–32]. However, chloride transport is poorly understood: there are conflicting reports on the expression of CFTR in both NCI-H441 cells [30, 33, 34] and A549 cells [34–38], and its role in ion/fluid homeostasis is debated [29, 39]. Furthermore, none of these studies have examined sodium and chloride transport in conjunction and there is no information about the paracellular and transcellular components of net transport. The properties of these cell line models have also not been compared to recent data from primary human cell cultures [11] and there is little information on how transport protein expression and function is affected by culture conditions that vary widely in cell seeding density [23, 40–43] and the nature and concentration of supplements [22, 27, 28, 40–42].

This study was designed to address these gaps. We carried out an in-depth analysis of the transport protein expression profile, cell morphology, barrier function and electrical properties of the two cell lines to determine their similarity to primary human alveolar epithelial cells. We then determined a standardised set of culture conditions that provide optimal cell phenotype and function and characterised the contributions of various channels to sodium and chloride transport under baseline and forskolin-stimulated conditions.

## Materials and Methods

### Cell culture and maintenance

NCI-H441 (also referred to as H441) cells (HTB-174) and A549 cells (CCL-185), were obtained from the American Type Culture Collection (ATCC, Manassas, USA) and grown in T-75 culture flasks in an atmosphere of 5% CO_2_ at 37°C. NCI-H441 cells and A549 cells were maintained in proliferation medium (RPMI 1640 (Catalog No. 21870–076) and F-12K respectively) containing 10% fetal bovine serum (FBS) and 1% penicillin-streptomycin (P/S) and 1% GlutaMAX (all from Thermo Fisher, Auckland, New Zealand). Cells were seeded onto 12-well Transwell inserts (Costar 3460, Corning, New York, USA) at a density of 100,000 cells/well in proliferation medium (0.5 ml in the apical and 1.5 ml in the basolateral chambers). The seeding day was defined as Day 0. Cells were allowed to attach for 24 hours before the medium in both chambers was replaced with polarization medium (see below). After the cells reached confluence (typically on day 3), the polarization medium was removed from the apical compartment, leaving the apical surface of the cells exposed to air (air-liquid culture). The polarization medium was made up of base medium RPMI 1640 or F-12K containing 4% FBS, 1% penicillin-streptomycin, 1% GlutaMAX, 1% insulin-transferrin-selenium (ITS, Thermo Fisher, Auckland, New Zealand), and 200 nM dexamethasone (Sigma, Auckland, New Zealand). Medium was changed every two days. Cell passages from 53–65 for the NCI-H441 cells and 82–95 for the A549 cells were used in this study.

### Transepithelial electrical resistance (TEER) and transepithelial potential difference (TEPD) measurements

TEER and TEPD measurements were used to evaluate the barrier function and active ion transport in the cell cultures. TEER is a measure of the permeability of the cell layer to ionic species while TEPD is a measure of the ability to generate and maintain ionic concentration gradients across the cell layer [44]. Pre-warmed Hanks' Balanced Salt Solution (HBSS) was added to the apical (0.5 ml) and basolateral (1.5 ml) chambers. Cells were equilibrated with HBSS for 10 minutes in the incubator in an atmosphere of 5% CO_2_ at 37°C. TEER and TEPD values were measured using an EndOhm-12 chamber voltohmmeter (World Precision Instruments, Sarasota, Florida, USA), following manufacturer’s instructions (available online at www.wpiinc.com/clientuploads/pdf/EndOhm_IMs.pdf). Briefly, the Transwell insert was placed in the voltohmmeter chamber and the electrical resistance/potential difference was measured. This was repeated using a blank insert without cells and the measurements were corrected by subtracting this value. The TEER was calculated by multiplying the measured resistance with the surface area of the inserts (1.12 cm^2^). TEPD values here are stated with the apical electrode as ground, i.e. a positive TEPD means that apical chamber is at a lower potential than the basolateral one.

### Permeability studies

Paracellular transport of sodium fluorescein (MW = 367 Da, Catalog No. F6377, Sigma, Auckland, New Zealand) was used to assess barrier integrity. To ensure that the integrity of the monolayer was maintained during the course of the experiment, TEER was measured before and after these studies. Before each experiment the culture medium was removed from the basolateral compartment and the monolayer was washed twice with warm HBSS (37°C). To measure the permeability in the apical-to-basolateral direction, 1.5 ml of pre-warmed HBSS was placed in the basolateral compartment. Cells were then returned to the incubator at 37°C for 30 minutes to equilibrate. Sodium fluorescein (0.5 ml of 10 μM in HBSS) was then added to the apical chamber. To measure the transport of sodium fluorescein over time, samples of 0.1 ml were taken from the basolateral compartment of each well every 30 minutes from 0 to 120 minutes, and replaced by equal amount of fresh warm HBSS. After confirming that the diffusion of sodium fluorescein was linear, samples were taken at 0, 30 and 60 minutes to measure the permeability. The fluorescence of the samples was measured in black, 96-well plates using a Fluoroskan fluorescence plate reader (Ascent FL, Thermo Scientific, Waltham, Massachusetts, USA) with excitation and emission wavelengths of 460 nm and 515 nm respectively. Fluorescein concentrations in the basolateral compartment were calculated and plotted as a function of time. Permeability coefficients P_app_ were calculated using the equation: P_app_ = ((dC/dt)V)/(AC_0_), where dC/dt is the slope of a linear fit to concentration vs. time plot, V is the volume of HBSS in the receiver chamber, A is the surface area of the membrane (1.12 cm^2^), and C_0_ is the concentration of fluorescein added to the donor (apical) compartment (10 μM) [22].

#### Quantitative real time PCR (qPCR)

RNA was extracted from cells using a PureLink^®^ RNA Mini Kit (Thermo Fisher, Auckland, New Zealand). RNA concentrations were measured using a Nanodrop 8000 (Thermo Scientific, Waltham, Massachusetts, USA). Genomic DNA was digested using post-column DNase I (Catalog AMPD1, Sigma, Auckland, New Zealand) treatment. Briefly, RNA (1 μg) was incubated with 1 unit of DNase I for 15 minutes at room temperature. The reaction was stopped by heating with 50 mM EDTA at 70°C for 10 minutes. Reverse transcription was carried out using the High Capacity cDNA Reverse Transcription Kit (Thermo Fisher, Auckland, New Zealand) following the manufacturer’s instructions. The cDNA (30 ng/reaction, 12 μl reaction volume) was amplified in an Applied Biosystems 7900HT Sequence Detection System (Thermo Fisher, Auckland, New Zealand) using QuantiTect SYBR Green PCR Kit (Biostrategy, Auckland, New Zealand) in 384 well microplates. The PCR amplification involved a single denaturing step of 95°C for 15 minutes, and then 45 cycles of 94°C for 15 seconds, 56°C for 30 seconds, and 72°C for 30 seconds. The thermal dissociation protocol was run at the end of the PCR to assess the specificity of products that were present in the reaction. RNA from three different batches of cells was analysed. Each reaction included three technical replicates and no-reverse transcriptase (no-RT) controls for each gene. Data were analysed using the software SDS 2.4 (Thermo Fisher, Auckland, New Zealand). *C*_*t*_ values ranged from 16 to 38 for most target genes. If signal was detected after 45 cycles these genes were considered undetectable. No signal was detected in the no-RT control samples except for four target genes (SP-A, β-ENaC GAPDH and Claudin-2). For the first three genes, *C*_*t*_ values for the no-RT controls were at least 15 cycles greater than the cDNA samples. In the case of Claudin-2, *C*_*t*_ values for the no-RT controls were 6 cycles greater. All primer sequences and amplicon sizes are listed in S1 Table. All of the primer pairs yielded a single peak on the thermal dissociation curve and a single band of the correct size on agarose gels (data not shown). GAPDH, TBP and β-actin were used as reference genes for nomalization. All three reference genes had GeNorm M < 1 and CV < 0.5 across samples from both cell lines and therefore meet published criteria for stable expression [45]. The relative expression of a target gene is expressed as

$$\Delta C_{t}=C_{t}^{ref}-\;C_{t}^{target}+\;20$$

where $C_{t}^{ref}$ is the mean *C*_*t*_ of the three reference genes. The addition of 20 makes the equation equal to log_2_(10^6^ × normalised gene expression) and makes the visual presentation of results more intuitive.

### Western Blotting

Cells were lysed in RIPA buffer (R0278, Sigma, Auckland, New Zealand) containing 1% protease inhibitor cocktail (P8340, Sigma, Auckland, New Zealand). Protein concentrations were determined using the DC^™^ Protein Assay (Bio-Rad, Auckland, New Zealand). Prior to SDS-PAGE samples were heated at 95°C for 5 minutes in 4 × Laemmli sample buffer (Bio-Rad, Auckland, New Zealand) and 2-mercaptoethanol (Sigma, Auckland, New Zealand). For detection of CFTR, samples were heated at 37°C for 15 minutes prior to SDS-PAGE to prevent its preciputation [46]. The airway epithelial cell line Calu-3 was used as a positive control for CFTR and to optimise the protocol. Protein samples (40 μg) were separated on 4–15% Mini-PROTEAN^®^ TGX^™^ precast polyacrylamide gels (Bio-Rad, Auckland, New Zealand) and transferred onto PVDF membranes (Bio-Rad, Auckland, New Zealand). The membranes were blocked in 5% skim milk in Tris-buffered saline with 0.1% Tween 20 for 1 hour at room temperature and then incubated with primary antibodies overnight at 4°C followed by the appropriate HRP-conjugated secondary antibody for 2 hours at room temperature. β-actin levels were quantitated in all samples using one of two β-actin antibodies–both gave similar results. All the antibodies used in this paper are listed in the S2 Table. Immunoreactive bands were visualized using Clarity^™^ Western ECL Substrate kit (Bio-Rad, Auckland, New Zealand) according to the manufacturer’s instructions and images were captured on an ImageQuant LAS 4000 (Fuji Film, Tokyo, Japan).

### Immunofluorescence microscopy

Cells in Transwell inserts were fixed with -20°C methanol for 5 minutes at 4°C. Cells were then washed three times with PBS, blocked with 1% bovine serum albumin in PBS (Thermo Fisher, Auckland, New Zealand) for 60 minutes at room temperature, and then incubated with primary antibodies overnight at 4°C. After three washes with PBS, cells were incubated with the appropriate fluorescence-conjugated secondary antibodies (S2 Table) for 2 hours at room temperature. Cell nuclei were counterstained with Hoechst 33342 (Thermo Fisher, Auckland, New Zealand) for 10 minutes at room temperature. A subset of samples was stained with Hoechst 33342 only to assess the effect of seeding density on the development of multiple layers of cells. Transwell membranes were then sliced into strips and mounted onto glass slides with the cell side up, and treated with ProLong^®^ Gold anti-fade reagent (Thermo Fisher, Auckland, New Zealand) overnight at 4°C with coverslips on top. Cells were imaged using a ZEISS LSM 710 confocal microscope with a 40x oil-immersion lens. Z stack scanning was done in an apical to basolateral direction with an interval of 0.5 μm. Laser settings were optimised to obtain the best signal for each sample. No-primary antibody controls did not exhibit any specific staining. Images were processed and analysed using ZEN 2011 software (black edition, Carl Zeiss, Germany).

### Inhibitor and agonist treatments

NCI-H441 cells were grown under the air-liquid culture condition until they reached the maximum resistance (Day 9)—only cells with TEER higher than 250 Ω cm^2^ were used. Cells were treated with amiloride (ENaC inhibitor), CFTR(inh)-172 (highly specific CFTR inhibitor), NPPB (a broad spectrum chloride channel inhibitor), or forskolin (cAMP agonist) in 0.5 ml HBSS on the apical side, or with ouabain (Na^+^-K^+^-ATPase inhibitor) in 1.5 ml HBSS on the basolateral side. All chemicals were used at a concentration of 10 μM. The duration of exposure was 5 minutes for amiloride, CFTR(inh)-172 and NPPB, and 10 minutes for forskolin and ouabain. All chemicals were supplied by Sigma (Auckland, New Zealand). TEPD was measured across the cell monolayer before and after treatment. Cells treated with HBSS were used as control.

Forskolin activates both ENaC and CFTR via an increase in intracellular cAMP. To study the individual contributions of ENaC and CFTR to forskolin stimulation, two protocols were followed. In one case, cells were treated with forskolin for 10 minutes to activate both ENaC and CFTR before adding amiloride, CFTR(inh)-172 or NPPB to the apical side. To study the forskolin response independent of ENaC activation, cells were treated with amiloride first before applying forskolin, CFTR(inh)-172 or NPPB.

### Statistical Analysis

To ensure reproducibility of the results, at least two independent experiments were performed using different batches of cells. In each experiment, TEER, TEPD and P_app_ were measured in at least 3 Transwells while qPCR, western blot and immunofluorescence studies were performed using 1 Transwell each. Each individual Transwell was treated as an independent sample for statistical analysis. Data were analysed using Student’s t-test for single comparisons and one-way or two-way ANOVA analysis followed by post-hoc Dunnett or Bonferroni tests for multiple comparisons. Analysis was carried out using the software package GraphPad Prism version 6.0. Results were considered significant at *P* < 0.05.

## Results

### NCI-H441 and A549 cells exhibit different TEER, TEPD and permeability properties

**Fig 1:**
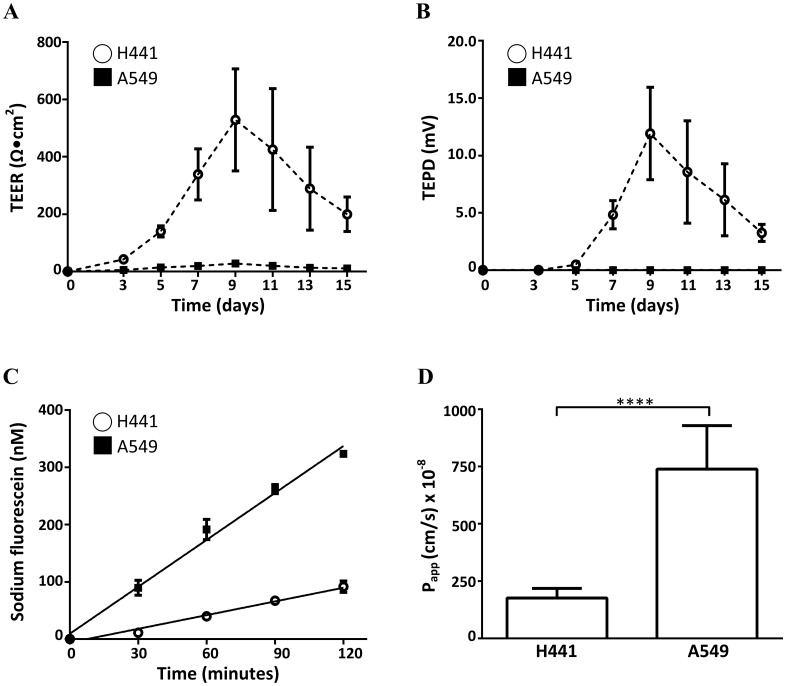
Barrier properties of H441 and A549 cells. (A) TEER and (B) TEPD across H441 cell and A549 cell monolayers measured in the culture period of 0–15 days (*n* = 9). (C) Time course (*n* = 6) and (D) apparent permeability (*n* = 11) of sodium fluorescein across H441 and A549 monolayers measured in the apical-to-basolateral direction on Day 9. Data are shown as mean ± SD; ****, *P* < 0.0001 by Student’s t- test.

TEER values for NCI-H441 cells under the air-liquid culture condition increased during the culture period to a maximum value of 529 ± 178 Ω cm^2^ on day 9 and remained higher than 250 Ω cm^2^ for 5 days (Fig 1A). In contrast, TEER values for A549 cells showed little change over the same time period with a peak of 28 ± 4 Ω cm^2^ on day 9 (Fig 1A). The TEPD values of NCI-H441 cells reached a maximum of 11.9 ± 4 mV on day 9 (Fig 1B). In contrast, TEPD values from A549 cells remained close to 0 mV over the same period (Fig 1B). As the maximum TEER and TEPD values of NCI-H441 cells and A549 cells were seen on day 9, this time point was selected for permeability measurements using the paracellular tracer sodium fluorescein. The permeability for the blank inserts was (2773 ± 76) ×10^−8^ cm/s (data not shown). The concentration vs. time plots were linear for both cell lines with R^2^ = 0.97 for NCI-H441 and R^2^ = 0.99 for A549 respectively (Fig 1C), indicating that the data are consistent with the passive diffusion model used for calculating permeability. The P_app_ values of NCI-H441 and A549 cells were (176 ± 42) ×10^−8^ cm/s and (738 ± 190) ×10^−8^ cm/s respectively (Fig 1D). Taken together, these data indicate that NCI-H441 cells form a tighter and less permeable epithelium than A549 cells with the ability to maintain transepithelial ion concentration gradients.

### NCI-H441 and A549 cells exhibit mRNA expression of type II markers, barrier components and ion channels

**Fig 2:**
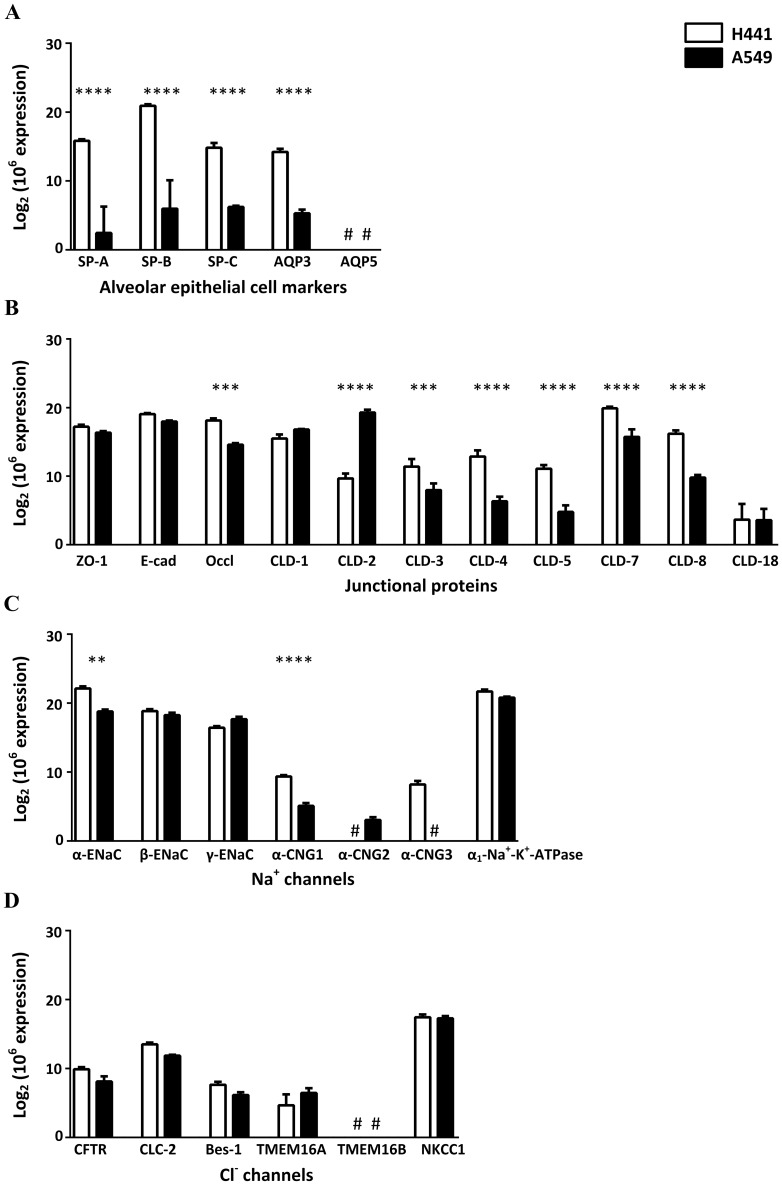
mRNA expression profiles of NCI-H441 and A549 cells. mRNA samples (n = 3) were obtained from three independent cultures of H441 cells and A549 cells. mRNA expression of (A) alveolar epithelial cell markers, (B) junctional proteins, (C) Na^+^ channels and (D) Cl^-^ channels were analysed by quantitative real-time PCR. Data are shown as mean ± SD; **, *P* < 0.01; ***, *P* < 0.001; ****, *P* < 0.0001 by two-way ANOVA analysis followed by post-hoc Bonferroni test. (#: target not detected.)

Gene expression profiles of alveolar epithelial cell markers, junctional proteins and transport proteins for Na^+^, Cl^-^ and water were determined in both cell lines by qPCR. Both NCI-H441 and A549 cells expressed the alveolar type II cell markers surfactant proteins SP-A, SP-B, SP-C and the water channel AQP3 but not the type I cell marker AQP5 (Fig 2A). NCI-H441 cells expressed higher levels of all the four type II cell markers compared with A549 cells. As for the junctional proteins, A549 cells expressed higher claudin-2, but NCI-H441 cells showed higher occludin and claudins-3, 4, 5, 7, 8 (Fig 2B). Both cell lines expressed transcripts encoding the α, β and γ subunits of ENaC, α_1_-Na^+^-K^+^-ATPase and the cyclic nucleotide-gated sodium channel α-CNG-1. NCI-H441 cells showed higher α-ENaC and α-CNG-1, but no detectable α-CNG-2. A549 cells showed no detectable α-CNG-3 (Fig 2C). Transcripts for the chloride channels CFTR, CLC-2, bestrophin-1 and TMEM16A, but not TMEM16B, were found in both cell lines. The Na^+^-K^+^-2Cl^-^ cotransporter NKCC1 was also expressed in both cell lines (Fig 2D). These results indicate that both cell lines express the key molecular components involved in ion and fluid transport, but there are differences in transcript levels. We next investigated if these differences translated to variations in protein expression.

### NCI-H441 and A549 cells exhibit differential expression of barrier and transport proteins at the protein level

**Fig 3:**
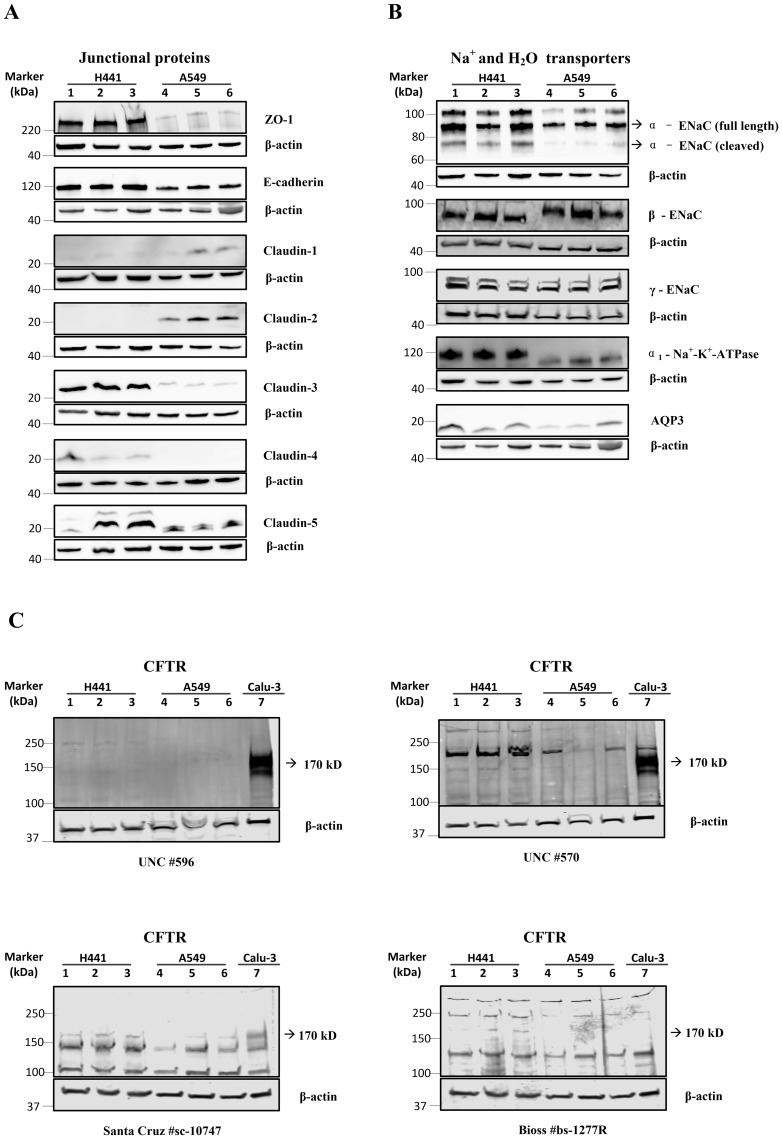
Protein expression profiles of NCI-H441 and A549 cells. Expression of (A) junctional proteins, (B) Na^+^ and water transporters and (C) CFTR was examined by Western blotting. Protein lysates from three independent cultures of H441 cells (Lane 1, 2 and 3) and A549 cells (Lane 4, 5 and 6) are tested (n = 3). In (C) Calu-3 cell lysates were used as positive control (Lane 7). Levels of actin expression were used to monitor protein loading. The size of the closest molecular weight marker to each target protein is shown.

The expression of junctional and transport proteins was detected by western blotting (Fig 3). Results were found to be consistent in three independent cultures within each cell line. ZO-1, E-cadherin and claudins-1, -3, -5 were expressed in both cell lines. NCI-H441 cells showed much higher expression of ZO-1 and claudin-3, -5, slightly higher expression of E-cadherin and lower expression of claudin-1 than the A549 cells (Fig 3A). However, claudin-2 and claudin-4 which were expressed at the mRNA level in both cells lines were not detected at the protein level in NCI-H441 and A549 cells, respectively (Fig 3A). Discrepancies between mRNA and the protein expression of claudins are not uncommon and have been reported for human corneal and conjunctival epithelium [47]. The difference may reflect low levels of the translation of claudin mRNAs into protein or rapid protein turnover. For the proteins that contribute to Na^+^, Cl^−^ and water transport, α_1_-Na^+^-K^+^-ATPase was more abundant in NCI-H441 cells than in A549 cells while ENaC subunits α, β, γ, and AQP3 were expressed at similar levels in both cell lines (Fig 3B). Three bands were found for α-ENaC in both NCI-H441 and A549 with sizes of approximately 104 kDa, 90kDa and 74 kDa cells. The lower two bands are likely to correspond to full length (uncleaved) and cleaved α-ENaC. The cleaved form is more abundant in NCI-H441 cells compared to A549 cells indicating higher α-ENaC activity.

Four different antibodies were used to detect CFTR protein in the cell lines (Fig 3C). Two antibodies (University of North Carolina UNC #570 and UNC #596) showed prominent bands for CFTR around 170 kDa in the positive control airway epithelial cell line Calu-3. The UNC #570, but not the UNC #596 antibody showed faint bands around the same size in A549 and NCI-H441 cells. A third antibody (Santa Cruz sc-10747) also showed bands around 150–170 kDa in all three cell lines; however the detection of multiple bands by this antibody makes it difficult to interpret the findings. The fourth antibody (Bioss bs1277R) showed bands at a lower molecular mass of around 120 kDa. Thus the expression of CFTR protein in A549 and NCI-H441 cells cannot be definitively ruled out. However it is likely that any expression is at a low level. This could explain why some studies have demonstrated that NCI-H441 cells [33] and A549 cells express CFTR, while others have reported that they do not [34, 36, 38].

### NCI-H441 and A549 cells form polarised epithelium under air-liquid culture condition

**Fig 4:**
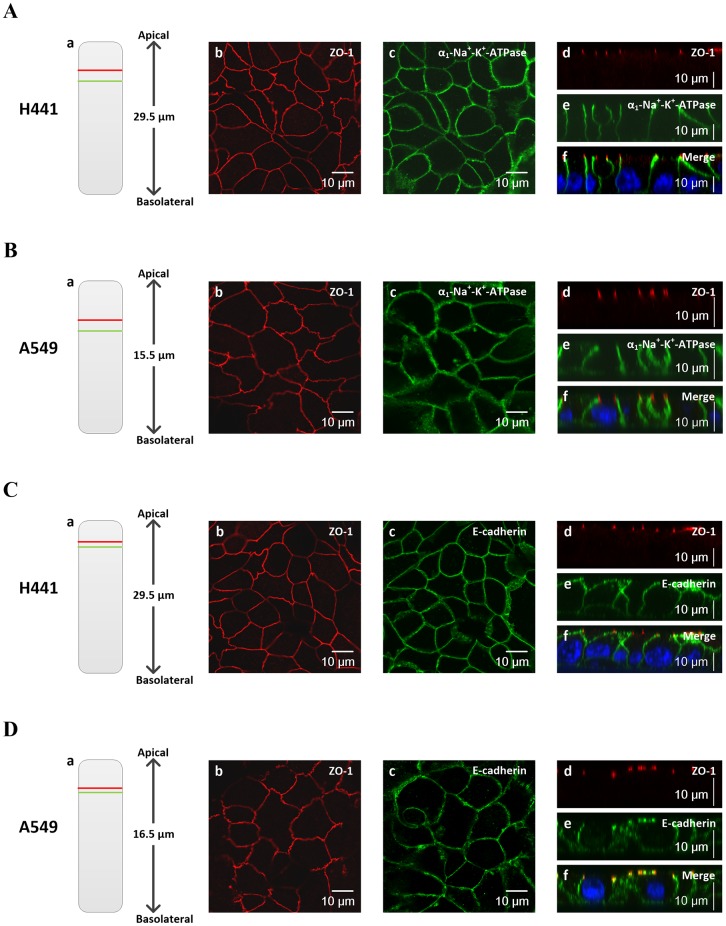
Immunostaining of H441 and A549 cells for ZO-1, E-cadherin and α_1_-Na^+^-K^+^-ATPase. (A, B) H441 cells and A549 cells were double stained for either ZO-1 and α_1_-Na^+^-K^+^-ATPase or (C, D) ZO-1 and E-cadherin. (a) The locations of the XY slices are shown in the schematic YZ section. Nuclei are stained blue. (b, c) XY and (d–f) YZ scans are shown. Representative images from two independent experiments were shown.

Cell layer morphology and expression of junctional proteins was visualised by confocal imaging following immunofluorescent staining. Digital reconstruction of confocal images showed that NCI-H441 cells had heights of around 29.5 μm while A549 cells were flatter with heights of around 16 μm. Lateral dimensions for both cells types were 5–20 μm (Fig 4A and 4B). The morphology was consistent with the cuboidal shape associated with type II cells. Immunofluorescence images showed continuous staining of ZO-1, E-cadherin and α_1_-Na^+^-K^+^-ATPase around cell borders in both NCI-H441 and A549 cells (XY sections, Fig 4A–4D, panels b and c). ZO-1 expression was confined to the apical extremity of the membrane while E-cadherin and α_1_-Na^+^-K^+^-ATPase were expressed more basolaterally (YZ sections, Fig 4A–4D, panels d-f). The segregation of the proteins to the apical and basolateral aspects of the membrane is an indicator of epithelial polarization [48]. Therefore, we can conclude that both NCI-H441 cells and A549 cells form a polarised monolayer in our cell culture model. However, consideration of all the data reveals that NCI-H441 cells develop better barrier function (higher TEER and lower permeability), more active ion transport (higher TEPD), and express higher levels of cleaved α-ENaC and α_1_-Na^+^-K^+^-ATPase that are necessary for Na^+^ transport. Hence we decided to focus on this cell line for characterizing the Na^+^ and Cl^−^ transport properties.

### Optimisation of cell culture conditions for the growth of a polarised NCI-H441 monolayer

**Fig 5:**
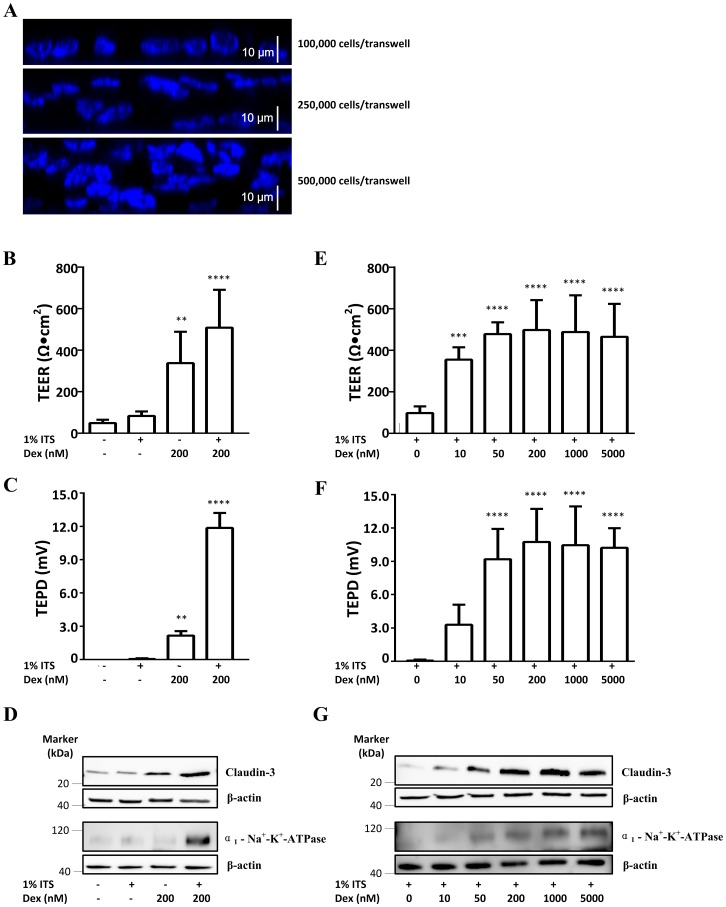
Optimisation of cell culture conditions. (A) Effect of seeding density on the development of H441 cell layers. Nuclei are stained blue. Cross-sections from YZ scans are shown. Effect of ITS on (B) TEER (n = 5), (C) TEPD (n = 5) and (D) protein expression in the presence and absence of dexamethasone. (E) Dose response of TEER (n = 7), (F) TEPD (n = 7) and (G) protein expression to dexamethasone in the presence of ITS. Protein expression data show representative images from two independent experiments. Data are shown as mean ± SD; **, *P* < 0.01; ***, *P* < 0.001; ****, *P* < 0.0001 using one-way ANOVA analysis followed by post-hoc Dunnett test.

The optimal culture conditions to yield best barrier function (TEER) and active ion transport (TEPD) in NCI-H441 cells were explored. Initial studies showed that TEER of over 300 Ω cm^2^ and TEPD of over 7 mV (Fig 1A and 1B) were consistently produced by using 200 nM dexamethasone with ITS. Thus this condition (basic medium supplemented with 4% FBS, 1% ITS and 200 nM dexamethasone) was used to determine the effect of seeding density. In our study, three seeding densities starting from 100,000 cells/transwell were explored. Cross-sectional images of Hoechst 33342-stained cell layers showed that a monolayer of cells developed at a seeding density of 100,000 cells/transwell, but multi-layers developed at higher densities of 250,000 and 500,000 cells/transwell (Fig 5A). Next, the role of ITS was determined at the seeding density of 100,000 cells/transwell. TEER (Fig 5B), TEPD (Fig 5C) and the expression of transport-related proteins (Fig 5D) were examined in the absence and presence of ITS at 0 nM and 200 nM dexamethasone respectively. Maximum TEER and TEPD were seen on Day 9 (data not shown). Using 1% ITS or 200 nM dexamethasone alone led to very low TEER, 83 ± 21 Ω cm^2^ and 337 ± 151 Ω cm^2^ respectively (Fig 5B), and very low TEPD, 0 and 2.2 ± 0.4 mV respectively (Fig 5C). Combination of 1% ITS and 200 nM dexamethasone resulted in significantly higher TEER 509 ± 182 Ω cm^2^ (Fig 5B) and TEPD 11.9 ± 1.3 mV (Fig 5C), which might be due to the robust expression of claudin-3 and α_1_-Na^+^-K^+^-ATPase (Fig 5D). As ITS was needed to induce moderate TEER and TEPD, the effect of dexamethasone (0–5000 nM) was further explored in the presence of 1% ITS (Fig 5E, 5F and 5G). As the concentration of dexamethasone increased from 0 nM to 200 nM, TEER, TEPD and expression of claudin-3 and α_1_-Na^+^-K^+^-ATPase also increased. Levels of dexamethasone exceeding 200 nM induced similar level of TEER (~ 500 Ω cm^2^, Fig 5E), TEPD (~ 11 mV, Fig 5F) and expression of claudin-3 and α_1_-Na^+^-K^+^-ATPase (Fig 5G). Dexamethasone and ITS had little effect on the expression of ZO-1, E-cadherin, claudins-1, 4, 5, α-ENaC and AQP3 (data not shown). In addition, 10 nM triiodo-L-thyronine (T3) in the presence of 1% ITS and 200 nM dexamethasone did not have a significant effect on TEER and TEPD (data not shown). These experiments indicated that cells at a seeding density of 100,000 cells/transwell cultured in the basic medium supplemented with 4% FBS, 1% ITS and 200 nM dexamethasone achieved a tight, polarised NCI-H441 cell monolayer and these conditions were used in subsequent experiments.

### ENaC is the major contributor to the transepithelial potential difference in NCI-H441 cells at baseline and forskolin-stimulated conditions

**Fig 6:**
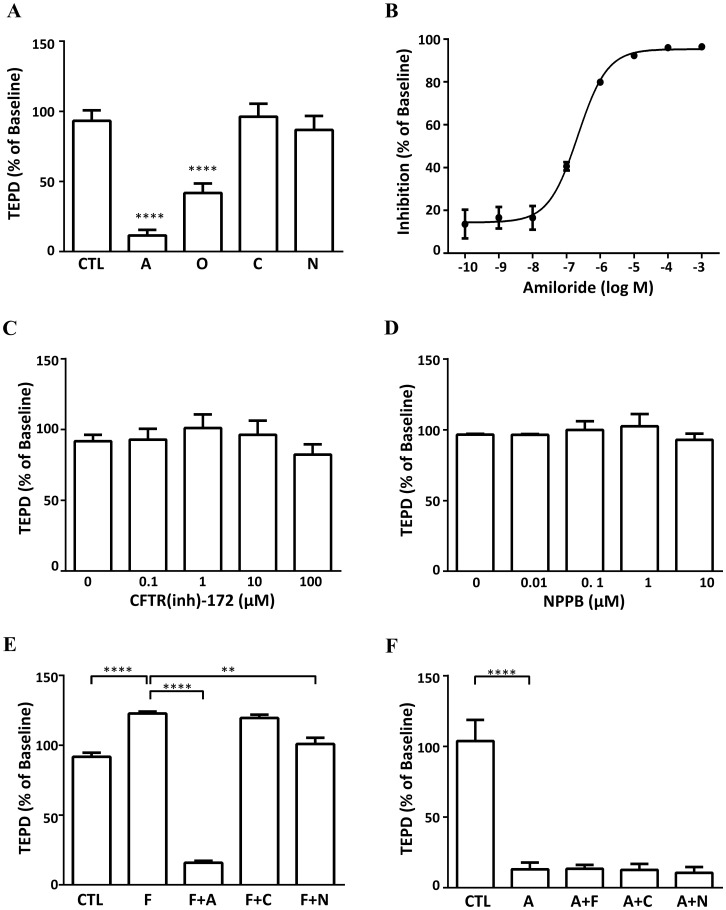
Na^+^ and Cl^−^ transport under baseline and stimulated conditions across H441 cells. (A) Response of TEPD to different perturbations. Each agent was used at a concentration of 10 μM (n = 13 for amiloride; n = 6 for ouabain, CFTR (inh)-172 and NPPB). Dose response of TEPD to: (B) ENaC inhibitor amiloride (n = 5), (C) CFTR inhibitor CFTR (inh)-172 (n = 6) and (D) broad spectrum chloride channel inhibitor NPPB (n = 6). (E) Effect of forskolin followed by amiloride, CFTR(inh)-172 or NPPB on TEPD (n = 9). (F) Effect of amiloride followed by forskolin, CFTR(inh)-172 or NPPB on TEPD (n = 7). (CTL: control; A: amiloride; O: ouabain; C: CFTR (inh)-172; N: NPPB; F: forskolin). Results are combined from three independent experiments. Data are shown as mean ± SD; **, *P* < 0.01; ****, *P* < 0.0001 using one-way ANOVA analysis followed by post-hoc Dunnett test.

Treatment with the ENaC inhibitor amiloride (10 μM) and the Na^+^-K^+^-ATPase inhibitor ouabain (10 μM) significantly reduced TEPD by 91% and 58% respectively, while the CFTR inhibitor CFTR(inh)-172 (10 μM) and the broad spectrum chloride channel inhibitor NPPB (10 μM) had no effect (Fig 6A). The results indicate that sodium absorption by ENaC is responsible for most of the baseline TEPD, that Na^+^-K^+^-ATPase extrudes the absorbed Na^+^ into the basolateral space and that Cl^-^ transport through CFTR or NPPB-sensitive Cl^-^ channels does not contribute to the TEPD at baseline. After washout of the inhibitors/agonist with HBSS, the TEPD returned to baseline levels indicating that the perturbation in TEPD is transient and is not a result of permanent inactivation of the channels (data not shown). Amiloride dose-response experiments indicated a maximum inhibition of 94% and a half maximal inhibitory concentration (IC50) of 217 nM (Fig 6B) which is similar to the value of ~ 500 nM reported in primary human Type II cells [11]. The amiloride-sensitive sodium channels (ENaC) are responsible for 94% of Na^+^ transport, which suggests that Na^+^ enters the cells primarily through ENaC, but other amiloride-insensitive sodium channels (such as CNGs, which were detected at mRNA level, see Fig 2C) may also participate in the Na^+^ transport. To check if the lack of response to chloride channel inhibition was a concentration effect, the two inhibitors were used in concentrations between 0.1 to 100 times their reported IC50 values for CFTR (1 μM for CFTR(inh)-172 [49] and 0.08 μM for NPPB [50]); the results indicated no effect on the TEPD (Fig 6C and 6D).

Finally the contributions of ENaC and Cl^-^ channels to ion transport following treatment with the cAMP agonist forskolin (10 μM) were determined. Forskolin treatment of control cells resulted in a 32% increase in TEPD (Fig 6E). Subsequent addition of amiloride reduced the forskolin-stimulated TEPD to 84% below the baseline value. Addition of CFTR(inh)-172 to forskolin-treated cells caused a small (~ 4%) decrease in TEPD that was not statistically significant while addition of NPPB resulted in a 15% decrease (*P* < 0.01). When cells were pre-treated with amiloride before adding forskolin no changes in TEPD were detected (Fig 6F). The data suggest that Na^+^ absorption via ENaC is the primary determinant of TEPD under baseline and forskolin-stimulated conditions; that Cl^-^ transport via CFTR or NPPB-sensitive channels is not detectable at baseline; and that NPPB-sensitive channels, but not CFTR secrete Cl^-^ following forskolin stimulation.

## Discussion

The main results of this work are: 1) NCI-H441 cells cultured at an air-liquid interface replicate the phenotypic and functional ion transport characteristics of primary cultures of human type II alveolar epithelial cells; 2) culture conditions strongly influence the morphological, phenotypic and functional characteristics of the model; and 3) the cells form an absorptive epithelium with ENaC-mediated sodium absorption and non-CFTR driven chloride motion as the primary pathways of ion transport.

The primary aim of this study was to establish and characterise a robust and reproducible *in vitro* model of the human alveolar epithelium for the study of ion and water transport. While primary cell cultures are the gold standard for such models, difficulties in obtaining and long term passaging of primary cells have limited their use. We therefore focussed on the human cell lines NCI-H441 and A549 that have been widely used as models of the alveolar epithelium [18–23, 26–30, 33, 39, 51, 52]. Our study further evaluated these two cell models and focused on several important areas not covered by these studies: comparison with primary cell culture models, effect of culture conditions on model phenotype and function and the individual contribution of transcellular and paracellular pathways to ion transport.

At the mRNA level both NCI-H441 and A549 cell types expressed type II cell markers, tight junction components and ion transport components (Fig 2). Levels of type II cell markers were higher in NCI-H441 cells and only these cells form a functional barrier characterised by a significant transepithelial electrical resistance and potential difference (Fig 1A–1D). The electrical resistance of NCI-H441 cells (529 ± 178 Ω cm^2^) is similar to those of primary type II cells (419 ± 168 Ω cm^2^) while the potential difference is higher (11.9 ± 4 mV vs 3.2 mV) [11]. In both cases the amiloride-sensitive component contributes > 90% to the baseline potential difference. This similarity is mirrored in the protein expression profiles. Primary human alveolar type II cells express junctional proteins ZO-1, claudins-3, -4, -5 and -18 and E-cadherin [53, 54]. In our study, protein expression of these components was detected in the NCI-H441 cells (except claudin-18, which was not tested). We also show for the first time that NCI-H441 cells express AQP3 and confirm that they do not express AQP5 [51]. The ion transport proteins α-ENaC, β-ENaC, γ-ENaC and α_1_-Na^+^-K^+^-ATPase were also detected in the NCI-H441 cells which is consistent with previous studies [11]. In our study CFTR was expressed at the mRNA level in both cell lines, but a definitive signal was not obtained at the protein level suggesting that it may only be expressed at low abundance in both cell types (Fig 3C). The results show that the NCI-H441 cell line has phenotypic and functional characteristics that are similar in many respects to human type II cells.

In contrast, the A549 cell line forms a leaky barrier with low electrical resistance and high paracellular permeability and does not develop a transepithelial potential difference (Fig 1A–1D). These results are consistent with previous studies that have found that A549 cells are incapable of forming functional tight junctions [18, 40, 50, 55]. The contrasting barrier functions between the two cell lines may be due to the differential expression of junctional proteins. The claudin family plays different roles in regulating paracellular permeability. Pore-forming claudins (such as claudin-2) increase the paracellular permeability to ions or water when overexpressed while seal-forming claudins (such as claudin-3, -4 and -5) decrease the paracellular permeability [3, 56]. At the protein level, NCI-H441 cells did not express pore-forming claudin-2 and expressed high levels of seal-forming claudin-3, -4, and -5. On the other hand, A549 cells expressed claudin-2, higher levels of claudin-1, lower levels of claudin-3 and -5, and no claudin-4. In addition, NCI-H441 cells also expressed higher levels of ZO-1 and E-cadherin.

A second aim of the study was to establish the effect of culture conditions on the properties of the *in vitro* model. Reported culture conditions for air-liquid culture of NCI-H441 cells vary widely in seeding density (20,000–5,000,000 cells/cm^2^) [23, 40–43], dexamethasone concentration (10–2000 nM) [22, 27, 40–42], and the use of supplements such as ITS and T3 [27, 28]. We examined the effect of these variables on the phenotypic and functional characteristics of the model. A previous study with a seeding density of 20,000 cells/cm^2^ observed the formation of a monolayer [40] while other studies have not characterised cell layer morphology [23, 27, 28, 33]. Using confocal imaging, we demonstrated for the first time that NCI-H441 cells seeded at 100,000 cells/transwell form a monolayer with columnar cells similar to the *in vivo* epithelium. On the contrary, higher densities lead to multiple cell layers growing on top of each other. Purely functional measurements such as TEER and P_app_ cannot characterise these structural features and we recommend that morphological characterisation of *in vitro* models should be carried out for applications in which the structure of the cell layer is important, e.g. drug absorption and pollutant transport in the alveolar epithelium.

Some studies have used charcoal stripped serum instead of FBS which could offer better reproducibility [28, 39]. In our experiments there was little variation in the model characteristics with different batches of FBS. Instead, dexamethasone and ITS supplementation were the most significant factors affecting TEER, TEPD and protein expression. We found that ITS and dexamethasone act synergistically and lead to higher TEER and TEPD. Changes were much smaller when either supplement was used in isolation. These changes correlated well with increases in the expression of Na^+^-K^+^-ATPase and claudin-3. The four-fold increase in TEPD when ITS and dexamethasone are used in conjunction (Fig 5C) mirrors the marked increase in the expression of Na^+^-K^+^-ATPase (Fig 5D). This is also seen in the dose response to dexamethasone where TEPD and Na^+^-K^+^-ATPase markedly increase as dexamethasone is increased up to 50 nM and then level off (Fig 5F and 5G). Similar trends are seen in the response of TEER and claudin-3. Consistent with these results, claudin-3 has been found to act as a sealing component of the tight junction to increase barrier function in epithelia from various organs and species (including humans) [53]. However, two studies in rat alveolar epithelial cells have found that overexpression of claudin-3 decreased TEER and barrier function [57, 58]. Whether these reflect species- and/or cell line-specific differences or result from interactions between the different claudins will require further investigation. Our results provide a rational basis for the choice of culture conditions in working with NCI-H441 cells and also suggest an important role for claudin-3 in regulating barrier function in the model.

The final aim of the study was to determine the pathways responsible for sodium and chloride transport in the NCI-H441 model. Gene expression of a number of sodium and chloride transport proteins was found in the NCI-H441 cells (Fig 2A) and inhibitor studies were used to identify the contributions of specific pathways. Amiloride treatment reduced the TEPD by a maximum amount of 94% with an IC50 of 217 nM. These values are similar to measurements in primary human type II cultures [11] and indicate that sodium is absorbed by NCI-H441 and that the transport is primarily transcellular and mediated by ENaC.

Next we examined chloride transport and found that both CFTR-specific and broad spectrum chloride channel inhibitors did not change TEPD (Fig 6C and 6D). Since Cl^-^ absorption is required for electroneutrality, the data suggest that Cl^-^ transport under baseline conditions occurs either via the paracellular pathway or through chloride channel(s) that are not inhibited by CFTR(inh)-172 or NPPB. Our measurements cannot distinguish between these possibilities. These findings are broadly consistent with published results: in two studies CFTR inhibition caused a small decrease (NCI-H441 cells [39]) or no change (primary human type II cells [59]) in fluid absorption implying that CFTR-mediated Cl^-^ absorption is not of primary importance while another study [11] found a small degree of CFTR-mediated Cl- secretion at baseline (~ 5% increase in TEPD following CFTR inhibition). In order to investigate the role of Cl^-^ channels under stimulated conditions, NCI-H441 cells were treated with the cAMP agonist forskolin that can activate both ENaC and CFTR. Forskolin induced an increase in TEPD that was abrogated by subsequent treatment with amiloride, consistent with activation of ENaC. Treatment with CFTR(inh)-172 following forskolin stimulation caused a small decrease in TEPD (~ 4% from peak) that was not statistically significant while treatment with NPPB caused a decrease of 15% indicating Cl^-^ secretion via non-CFTR channels under this condition. Forskolin-induced ENaC activation will depolarise the apical membrane and reduce the driving force for Cl^-^ secretion. We tested if secretion could be enhanced by blocking ENaC with amiloride prior to the addition of forskolin. Surprisingly, no changes in TEPD were observed (Fig 6F). Similar results have been reported in primary human type II cells in which forskolin stimulation following amiloride pre-treatment did not elicit a response (Fig 6A) [59] which was ascribed to the lack of a significant driving force for transepithelial Cl^-^ transport under baseline and amiloride-treated conditions. We suggest that our results reflect differences in basolateral Cl^-^ influx via NKCC1 or other Cl^-^ channels. When ENaC is activated by forskolin treatment, increased intracellular Na^+^ provides the impetus for Cl^-^ entry across the basolateral membrane and subsequent secretion across the apical membrane. With ENaC inhibition the impetus for basolateral Cl^-^ entry (and hence apical secretion) is removed. The results indicate that Cl^-^ is secreted, not absorbed, via non-CFTR, NPPB-sensitive channels under cAMP stimulation in our model.

## Conclusions

In conclusion, we have carried out detailed characterization of a robust, reproducible *in vitro* model of the human alveolar epithelium and established the culture conditions to produce optimal morphological, phenotypic and functional characteristics for the study of alveolar fluid and ion transport. The model will be useful for studying regulatory mechanisms and pathways involved in alveolar fluid homeostasis.

## Supporting Information

S1 Table Primer list. Information about primers used in the qPCR assay is listed in S1 Table including target genes, amplicon sizes, primer sequences, whether primers span introns and literature source for the primers if applicable.
(DOCX)

S2 Table Antibody list. Information about antibodies used for western blotting and immunofluorescence microscopy is listed in S2 Table, including target proteins, suppliers and catalog numbers.
(DOCX)
