# Supplementary material for: An Optimised Human Cell Culture Model for Alveolar Epithelial Transport
Source: PLoS One. 2016 Oct 25;11(10):e0165225. doi: 10.1371/journal.pone.0165225 (PMC5079558; doi:10.1371/journal.pone.0165225)
Supplement: S1 Table — Information about primers used in the qPCR assay is listed in S1 Table including target genes, amplicon sizes, primer sequences, whether primers span introns and literature source for the primers if applicable. (DOCX) [file pone.0165225.s001.docx]

**S1 Table. Primer list**

| Target gene | Amplicon  Size (bp) | Primer sequence  (5’🡪3’) | Intron spanning | Reference |
| --- | --- | --- | --- | --- |
| ZO-1 | 122 | Forward:  GCGGTCAGAGCCT TC TGATC  Reverse:  CATGCTTTACAGGAGTTGAGACAG | Yes | [[1](#_ENREF_1)] |
| E-cadherin | 247 | Forward:  GCTGGACCGAGAGAGTTTCC  Reverse:  GGTGTATACAGCCTCCCACG | Yes | Designed using NCBI primer designing tool [[2](#_ENREF_2)] |
| Occludin | 189 | Forward:  TCAGGGAATATCCACCTATCACTTCAG  Reverse:  CATCAGCAGCAGCCATGTACTCTTCAC | Yes | [[3](#_ENREF_3)] |
| Claudin-1 | 233 | Forward:  CCGTTGGCATGAAGTGTATG  Reverse:  AAGGCAGAGAGAAGCAGCAG | Yes | [[4](#_ENREF_4)] |
| Claudin-2 | 91 | Forward:  ACCTGCTACCGCCACTCTGT  Reverse:  CTCCCTGGCCTGCATTATCTC | No | [[5](#_ENREF_5)] |
| Claudin-3 | 247 | Forward:  ACCGCCAATACTTGACCAC  Reverse:  TTTTTTTTTTTTTTTTTGCAAAACG | No | [[6](#_ENREF_6)] |
| Claudin-4 | 187 | Forward:  GGACAGCTTCACCCTTGG  Reverse:  TTTTTTTTTTTTTTTCCTGTGCA | No | [[6](#_ENREF_6)] |
| Claudin-5 | 108 | Forward:  GAGCAGCCCCTGTGAAGATT  Reverse:  GTCTCTGGCAAAAAGCGGTG | No | Design by NCBI primer designing tool [[2](#_ENREF_2)] |
| Claudin-7 | 84 | Forward:  ATCCCTACCAACATTAAGTATGAGTTTG  Reverse:  TGCACCTCCCAGGATGACTAG | Yes | [[7](#_ENREF_7)] |
| Claudin-8 | 71 | Forward:  TGCTGACGGCTGGAATCAT  Reverse:  ATTGGCAACCCAGCTCACA | No | [[7](#_ENREF_7)] |
| Claudin-18 | 81 | Forward:  TCATGTTCATTGTCTCAGGTCTTTG  Reverse:  ACATCCAGAAGTTAGTCACCAGCAT | Yes | [[7](#_ENREF_7)] |
| α-ENaC | 101 | Forward:  CTTTGGCATGATGTACTGGCA  Reverse:  GGAAGACGAGCTTGTCCGAGT | No | [[8](#_ENREF_8)] |
| β-ENaC | 101 | Forward:  GAGCCCTGCAACTACCGGA  Reverse:  GCCGAAGGAAGTGCCTTCTC | Yes | [[8](#_ENREF_8)] |
| γ-ENaC | 215 | Forward:  ACAGCCAGCCTCTACCTCCT  Reverse:  AACAGGCAGCAACCACTTCT | Yes | [[9](#_ENREF_9)] |
| α-CNG1 | 191 | Forward:  TGCTGATTGTGAAGCTGGTC  Reverse:  AGTAGCTGCCATCGCTCAAT | No | [[10](#_ENREF_10)] |
| α-CNG2 | 200 | Forward:  CTCCGTCCTCAGGTCTTCAG  Reverse:  CGTCGATTGCCCATTTTACT | No | [[10](#_ENREF_10)] |
| α-CNG3 | 239 | Forward:  ATCACCTCGTGTGTTCTTTGG  Reverse:  GGCTGTCTTTGAATCTCTTTG | No | [[11](#_ENREF_11)] |
| α_1_-Na^+^-K^+^-ATPase | 101 | Forward:  AGTTGCAGGAGATGCCTCTGA  Reverse:  TCTCGACGATTTTGGCGTATC | No | [[8](#_ENREF_8)] |
| CFTR | 103 | Forward:  TCCTCTCGTTCAGCAGTTTC  Reverse:  GAACACAGGATAGAAGCAATGC | Yes | Hs.PT.58.3365414; predesigned primers from Integrated DNA Technologies |
| NKCC1 | 132 | Forward:  TTTGTAAGAGGAGGAGGAGCA  Reverse:  TTCTGCAAATCCAACCACAT | Yes | [[12](#_ENREF_12)] |
| CLC2 | 234 | Forward:  AGTGGGAGGAGCAGCAACTA  Reverse:  GGACTTTCACACCCTGTGCT | Yes | [[10](#_ENREF_10)] |
| Bestrophin-1 | 130 | Forward:  CTAATGCCCGCTTAGGCTCC  Reverse:  GCCAGCCTATAAATAAAGCGGAT | Yes | [[13](#_ENREF_13)] |
| TMEM16A | 289 | Forward:  AAGCTCATCCGCTACCTGAA  Reverse:  CGATGTCTTTGGCTCTGACA | Yes | [[10](#_ENREF_10)] |
| TMEM16B | 298 | Forward:  TCGAAACATCCAGAGCAGTG  Reverse:  CAATGACAAAAGCGTTGCTG | Yes | [[10](#_ENREF_10)] |
| SP-A | 368 | Forward:  TTGGAGCCTGAAAAGAAGGA  Reverse:  GGCTTGGAGCTCCTCATCTA | Yes | [[10](#_ENREF_10)] |
| SP-B | 74 | Forward:  TGAGGACATCGTCCACATCC  Reverse:  CCAGGAACTTCCTCATCGTGT | Yes | [[14](#_ENREF_14)] |
| SP-C | 197 | Forward:  AGCCAGAAACACACGGAGATGGTT  Reverse:  ATCTTCATGATGTAGCAGCAGGTGCC | Yes | [[15](#_ENREF_15)] |
| AQP3 | 88 | Forward:  GAGATGCTCCACATCCGCTA  Reverse:  AGCCACAGCCAAACATCACC | Yes | Design by NCBI primer designing tool [[2](#_ENREF_2)] |
| AQP5 | 168 | Forward:  CAACAACAACACAACG  Reverse:  TAGATTCCGACAAGGT | Yes | [[16](#_ENREF_16)] |
| β-actin | 202 | Forward:  CACAGAGCCTCGCCTTTGCC  Reverse:  TGACCCATGCCCACCATCAC | Yes | [[17](#_ENREF_17)] |
| GAPDH | 216 | Forward:  CTCCTCTGACTTCAACAGCGACA  Reverse: GAGGGTCTCTCTCTTCCTCTTGT | Yes | [[17](#_ENREF_17)] |
| TBP | 135 | Forward:  GCTGTTTAACTTCGCTTCCGCTG  Reverse: GGTGTTCTGAATAGGCTGTGGGG | Yes | [[17](#_ENREF_17)] |

**References**

1. Stewart CE, Torr EE, Mohd Jamili NH, Bosquillon C, Sayers I. Evaluation of differentiated human bronchial epithelial cell culture systems for asthma research. Journal of allergy. 2012;2012:943982. doi: 10.1155/2012/943982. PubMed PMID: 22287976; PubMed Central PMCID: PMC3263641.

2. Ye J, Coulouris G, Zaretskaya I, Cutcutache I, Rozen S, Madden TL. Primer-BLAST: a tool to design target-specific primers for polymerase chain reaction. BMC bioinformatics. 2012;13:134. doi: 10.1186/1471-2105-13-134. PubMed PMID: 22708584; PubMed Central PMCID: PMC3412702.

3. Qin LH, Huang W, Mo XA, Chen YL, Wu XH. LPS Induces Occludin Dysregulation in Cerebral Microvascular Endothelial Cells via MAPK Signaling and Augmenting MMP-2 Levels. Oxid Med Cell Longev. 2015;2015:120641. doi: 10.1155/2015/120641. PubMed PMID: 26290681; PubMed Central PMCID: PMCPMC4531183.

4. Neuhaus W, Samwer F, Kunzmann S, Muellenbach RM, Wirth M, Speer CP, et al. Lung endothelial cells strengthen, but brain endothelial cells weaken barrier properties of a human alveolar epithelium cell culture model. Differentiation; research in biological diversity. 2012;84(4):294-304. doi: 10.1016/j.diff.2012.08.006. PubMed PMID: 23023065.

5. Moldvay J, Jackel M, Paska C, Soltesz I, Schaff Z, Kiss A. Distinct claudin expression profile in histologic subtypes of lung cancer. Lung cancer. 2007;57(2):159-67. doi: 10.1016/j.lungcan.2007.02.018. PubMed PMID: 17418912.

6. Hewitt KJ, Agarwal R, Morin PJ. The claudin gene family: expression in normal and neoplastic tissues. BMC Cancer. 2006;6(1). doi: 10.1186/1471-2407-6-186.

7. Lameris AL, Huybers S, Kaukinen K, Makela TH, Bindels RJ, Hoenderop JG, et al. Expression profiling of claudins in the human gastrointestinal tract in health and during inflammatory bowel disease. Scandinavian journal of gastroenterology. 2013;48(1):58-69. doi: 10.3109/00365521.2012.741616. PubMed PMID: 23205909.

8. Shlyonsky V, Goolaerts A, Van Beneden R, Sariban-Sohraby S. Differentiation of epithelial Na+ channel function. An in vitro model. The Journal of biological chemistry. 2005;280(25):24181-7. doi: 10.1074/jbc.M413823200. PubMed PMID: 15817472.

9. Araki I, Du S, Kamiyama M, Mikami Y, Matsushita K, Komuro M, et al. Overexpression of epithelial sodium channels in epithelium of human urinary bladder with outlet obstruction. Urology. 2004;64(6):1255-60. doi: 10.1016/j.urology.2004.06.064.

10. Bove PF, Grubb BR, Okada SF, Ribeiro CM, Rogers TD, Randell SH, et al. Human alveolar type II cells secrete and absorb liquid in response to local nucleotide signaling. The Journal of biological chemistry. 2010;285(45):34939-49. doi: 10.1074/jbc.M110.162933. PubMed PMID: 20801871; PubMed Central PMCID: PMC2966108.

11. Duricka Deborah L, Brown RL, Varnum Michael D. Defective trafficking of cone photoreceptor CNG channels induces the unfolded protein response and ER-stress-associated cell death. Biochemical Journal. 2012;441(2):685-96. doi: 10.1042/bj20111004.

12. Ibla JC, Khoury J, Kong T, Robinson A, Colgan SP. Transcriptional repression of Na-K-2Cl cotransporter NKCC1 by hypoxia-inducible factor-1. American journal of physiology Cell physiology. 2006;291(2):C282-9. doi: 10.1152/ajpcell.00564.2005. PubMed PMID: 16571862.

13. Gamm DM, Melvan JN, Shearer RL, Pinilla I, Sabat G, Svendsen CN, et al. A novel serum-free method for culturing human prenatal retinal pigment epithelial cells. Investigative ophthalmology & visual science. 2008;49(2):788-99. doi: 10.1167/iovs.07-0777. PubMed PMID: 18235029.

14. Boggaram V, Chandru H, Gottipati KR, Thakur V, Das A, Berhane K. Transcriptional regulation of SP-B gene expression by nitric oxide in H441 lung epithelial cells. American journal of physiology Lung cellular and molecular physiology. 2010;299(2):L252-62. doi: 10.1152/ajplung.00062.2010. PubMed PMID: 20418387; PubMed Central PMCID: PMC2928609.

15. Lin C, Song H, Huang C, Yao E, Gacayan R, Xu SM, et al. Alveolar type II cells possess the capability of initiating lung tumor development. PloS one. 2012;7(12):e53817. doi: 10.1371/journal.pone.0053817. PubMed PMID: 23285300; PubMed Central PMCID: PMC3527621.

16. Adamzik M, Frey UH, Bitzer K, Jakob H, Baba HA, Schmieder RE, et al. A novel-1364A/C aquaporin 5 gene promoter polymorphism influences the responses to salt loading of the renin-angiotensin-aldosterone system and of blood pressure in young healthy men. Basic Research in Cardiology. 2008;103(6):598-610. doi: 10.1007/s00395-008-0750-z.

17. Kaszubowska L, Wierzbicki PM, Karsznia S, Damska M, Slebioda TJ, Foerster J, et al. Optimal reference genes for qPCR in resting and activated human NK cells--Flow cytometric data correspond to qPCR gene expression analysis. J Immunol Methods. 2015;422:125-9. doi: 10.1016/j.jim.2015.04.013. PubMed PMID: 25914089.
