# Supplementary material for: An Optimised Human Cell Culture Model for Alveolar Epithelial Transport
Source: PLoS One. 2016 Oct 25;11(10):e0165225. doi: 10.1371/journal.pone.0165225 (PMC5079558; doi:10.1371/journal.pone.0165225)
Supplement: S2 Table — Information about antibodies used for western blotting and immunofluorescence microscopy is listed in S2 Table, including target proteins, suppliers and catalog numbers. (DOCX) [file pone.0165225.s002.docx]

**S2 Table. Antibody list**

| **Target protein** | **Supplier** | **Catalog number/name** |
| --- | --- | --- |
| ZO-1 | Thermo Fisher (Auckland, New Zealand) | 40-2200 |
| E-cadherin | Thermo Fisher (Auckland, New Zealand) | 13-1700 |
| Claudin-1 | Thermo Fisher (Auckland, New Zealand) | 51-9000 |
| Claudin-2 | Thermo Fisher (Auckland, New Zealand) | 51-6100 |
| Claudin-3 | Thermo Fisher (Auckland, New Zealand) | 34-1700 |
| Claudin-4 | Thermo Fisher (Auckland, New Zealand) | 32-9400 |
| Claudin-5 | Thermo Fisher (Auckland, New Zealand) | 34-1600 |
| Claudin-5 | Thermo Fisher (Auckland, New Zealand) | 34-2500 |
| α-ENaC | Santa Cruz (Dallas, Texas, USA) | sc-22239 |
| α_1_-Na^+^-K^+^-ATPase | Millipore (Billerica, Massachusetts, USA) | 05-369 |
| CFTR | Bioss (Woburn, Massachusetts, USA) | bs-1277R |
| CFTR | Santa Cruz (Dallas, Texas, USA) | sc-10747 |
| CFTR | The University of North Carolina at Chapel Hill (Chapel Hill, North Carolina, United States) | 570 |
| CFTR | The University of North Carolina at Chapel Hill (Chapel Hill, North Carolina, United States) | 596 |
| AQP3 | Santa Cruz (Dallas, Texas, USA) | sc-20811 |
| AQP5 | Santa Cruz (Dallas, Texas, USA) | sc-28628 |
| β-actin | Abcam (Cambridge, Massachusetts, USA) | ab8227 |
| β-actin | Gene Script (Piscataway, New Jersey, USA) | A00702-100 |
| β-actin | Thermo Fisher (Auckland, New Zealand) | PA1-183 |
| donkey anti-goat IgG-HRP | Santa Cruz (Dallas, Texas, USA) | sc-2020 |
| goat anti-rabbit IgG-HRP | Santa Cruz (Dallas, Texas, USA) | sc-2004 |
| goat anti-mouse IgG-HRP | Sigma (Auckland, New Zealand) | A4416 |
| Alexa Fluor® 488 goat anti-mouse | Thermo Fisher (Auckland, New Zealand) | A11001 |
| Alexa Fluor® 594 goat anti-rabbit | Thermo Fisher (Auckland, New Zealand) | A11012 |
